# A meta-analysis of peer-assisted learning on examination performance in clinical knowledge and skills education

**DOI:** 10.1186/s12909-022-03183-3

**Published:** 2022-03-05

**Authors:** Yanrui Zhang, Mark Maconochie

**Affiliations:** 1 Nanchang University Queen Mary School, Nanchang University, Room 215 Admin Building No. 1299 Xuefu Street, 330031 Nanchang, China; 2 School of Biological and Chemical Sciences, Queen Mary University of London, Mile End Road, E1 4NS London, UK

**Keywords:** Peer-assisted learning, Medical student education, Active learning, Clinical skills teaching

## Abstract

**Background:**

Peer-assisted learning is a method of active learning that is gaining traction throughout higher education. In the medical curriculum, peer-assisted learning has been the subject of independent studies collecting various types of data. However, an overall analysis of those studies providing objective measurements of the influence of peer-assisted learning could be particularly useful for teachers and students alike in a knowledge-heavy curriculum such as medicine. In this study we set out to analyse the efficacy of peer-assisted learning on medical students’ learning of clinical knowledge and skills that is assessed through some objective examination, and thereby define whether such approaches have a reproducible benefit for inclusion in the medical curriculum.

**Methods:**

Databases including Pubmed, Embase and Science Direct were searched for relevant studies containing randomized controlled trials (RCTs) of peer-assisted learning published before July 29th ,2020. A meta-analysis was performed by using RevMan 5.3 software.

**Results:**

Thirteen studies involving 2,003 medical students were analyzed for clinical knowledge and skills gains that included some objective measurement of learning. The results of this meta-analysis indicated that considering all these studies together, peer-assisted learning leads to improvements in clinical knowledge and skills learning for medical students compared with traditional teacher-led passive learning. One study was found likely to be a source of significant heterogeneity, and when this was removed from the meta-analysis, the pooled effect was no longer statistically significant.

**Conclusions:**

Peer-assisted learning can be an effective method of learning applied to medical student education. Active learning through peer-assisted learning should be seen as complementary to teacher-led approaches. Two of the individual studies on peer-assisted learning show a statistically significant benefit on examination performance compared to the other studies considered, that either show negligible benefits or at worst no detriment in learning. This highlights the need for more high-quality and focused randomized control trials to identify those critical parameters that lead to improved student learning using such approaches.

## Background

Traditional lecture-based teaching is the predominant educational strategy widely used and praised by many teachers and students in higher education across the world [1, 2]. It was long considered to be the best route to directly transfer knowledge to students [3]. However, the effectiveness of this approach is increasingly being questioned as this didactic teaching methodology is a passive, surface approach, that requires little commitment from students in their learning [4]. Further, it has been argued that the traditional lecture alone is inadequate and ineffective for current educational strategies, due to the passive nature and limit of students’ interactions [2, 5, 6]. Didactic delivery aims to transmit knowledge to students without any feedback and minimal interaction. This teaching strategy rarely mobilizes students’ initiative and is unable to inspire their creativity in the learning process. Traditional lectures thus tend to be relatively unsuccessful at initiating higher order thinking in students. This weakness has been exacerbated in recent years with the growth of the student population and concomitant class sizes, led by the demands of society worldwide to have a better educated workforce. An increase in class size leads to even fewer teacher-student classroom interactions [4].

Such passive learning strategies are in stark contrast to active learning, which is designed to stimulate student learning through performing tasks that directly engage them with knowledge acquisition and understanding. Furthermore, active learning arouses students’ enthusiasm to learn, promotes interactions between students [2, 7] and reinforces student-teacher interactions. Much research proposes that active learning is far more effective than the traditional lecture for deeper student learning [4, 8–18]. One active learning strategy is peer-assisted learning, defined as learning through matched-status individuals from “similar social groupings who are not professional teachers” [4, 9]. Topping [4] concluded that peer-assisted learning works particularly well when used alongside traditional lectures, and also is of benefit to the teacher as it allows for rapid feedback from students on their learning experience and depth.

However, whilst there is good evidence suggesting that peer-assisted learning is effective for students in general to develop life-long autonomous learning habits, most studies that look at the effectiveness of peer-assisted learning are qualitative. There are only a relatively small number of independent quantitative studies that provide objective statistical data and furthermore, few meta-analyses that attempt to quantitatively analyse the effect of peer-assisted learning. Balta [2] carried out a meta-analysis on peer-assisted learning across higher education that illustrated the positive effects on learning in addition to improvements in student achievement. Within the medical curriculum, a recent meta-analysis in 2020 [19] found significant effectiveness of peer-assisted learning, but this contrasts with an earlier meta-analysis in 2016 [20] that found no significant difference between peer-led and faculty teaching. There are several studies that in general show the positive effects for students of peer-assisted learning (for recent reviews see [12, 21]), where performance across a range of subjects in the medical curriculum have been examined to give an overall picture of the benefits. However, it is possible that peer-assisted learning may prove more beneficial in some specific subject areas in the medical curriculum. Given the potential for peer-assisted learning to be particularly beneficial to medical students, and the differing conclusions of previous, more general meta-analyses, this study was initiated to carry out a meta-analysis to systematically analyse the use of peer-assisted learning in clinical skills and knowledge teaching and learning. The meta-analysis is focussed on those studies where learning outcomes are objectively measured by some form of examination, given the importance of assessments as a driver to student learning.

## Methods

### Search strategy

A systematic search of the published literature was performed using Pubmed, Embase and Science Direct databases up to July 29th, 2020. Search terms used in literature searching were (“peer assisted learning” OR “PAL” OR “peer learn” OR “peer tutor” OR “peer teach”) AND (“traditional teaching” OR “faculty” OR “expert” OR “instructor” OR “staff” OR “tutor”). The specific search term “medical” was not used to ensure a sufficient pool of studies was recovered to apply the study selection procedure documented below.

### Study selection

The selection of research studies was performed following the PRISMA statement [22]. Studies were selected for inclusion in this meta-analysis only when they met the specific inclusion criteria detailed below. A hierarchical literature screen was carried out first through analysing article titles and excluding obviously irrelevant publications, and further delimited through deeper scrutiny of abstracts and the full text to identify papers in the appropriate subject area. All potentially eligible papers were retrieved and examined in full without bias to their conclusions in being selected. The aim of the database search and study selection procedure was to collect randomized-controlled trials of medical students receiving peer-assisted learning and traditional teacher-led learning in clinical skills and knowledge subjects.

*Inclusion criteria* for papers considered in the meta-analysis: (1) Study type: Randomized Controlled Trial; (2) Population: Medical students. (There was no limit on gender, age, race and nationality); (3) Intervention: the experimental group used peer-assisted learning to teach students, the control group used traditional teacher-led learning. Note that peer-assisted learning should conform to the definition given by Topping of “the acquisition of knowledge and skill through active helping and supporting among status equal or matched companions” [23]. Cooperative learning and peer-mentoring terms were not included; (4) Outcomes: clinical knowledge and clinical skills gain was measured by some form of objective examination after the intervention to assess whether the intervention led to any change in assessed learning outcome. Only randomised controlled trials were used in the meta-analysis as these provide the highest quality of evidence with quantitative outputs.

#### Exclusion criteria:(NOTE wrong level of subheading: Please use same font colour(black) and font size as for inclusion criteria subheading and italics as in 6 lines aboves)

(1) The reports were not available in English; (2). Studies with incomplete data sets; (3) Duplicate studies arising from different database searches; (4) Studies that consisted of subjective measurements of learning only such as student questionnaires, focus groups etc. Although these studies were excluded from the meta-analysis, some were used to provide useful and important commentary in the discussion.

### Data extraction and quality assessment

The following key information was collated during data extraction: basic information of the reported research, for instance, details of the first author, nature of intervention, academic level of student and peer-tutor, and nature/level of control teacher, type of objective assessments used and intervention subject area, whether any peer-training and its nature was offered, and whether the study reported any statistical support of the findings. The quality of the individual studies included in this meta-analysis was assessed according to Cochrane Collaboration’s tool for assessing risk of bias [24, 25].

### Statistical analysis

Statistical analysis was performed to minimize the risk of bias using RevMan 5.3 software. Parametric variables were presented as mean ± standard deviation (SD). The outcome of continuous variables used standardized mean difference (SMD) of exam scores with 95% confidence intervals (CIs) as the effect size. The level of significance for the meta-analysis was set as α = 0.05, i.e., a probability of *P* < 0.05 was judged statistically significant. Heterogeneity among the results from the different studies was detected by using the chi-square test (α = 0.1) together with calculating the *I²* statistic. The *I²* value gives an indication of the level of variation due to heterogeneity rather than due to chance [26]. *I²* was calculated using the formula *I²=*(Q-df)/Q×100%, where Q is the Cochran heterogeneity statistic and df the degrees of freedom, equivalent to the number of studies included in the meta-analysis minus one. A value of *I²* greater than 50% represents high heterogeneity amongst the data, and a value below 50% low or moderate heterogeneity [24, 26].

To analyse heterogeneity between studies, an influence analysis was carried out using the metaninf method. As this meta-analysis demonstrated a statistically significant level of heterogeneity (formally >50% represents substantial heterogeneity), a random-effect model for the meta-analysis was used. Cochrane collaboration’s tool was used to analyse the risk of bias of this meta-analysis. Revman was used to produce a risk assessment summary for the overall study group as well as to investigate the risk of bias for the individual studies. Further analysis of bias was investigated using a funnel plot to investigate publication bias. In order to test for significance for publication bias, the Egger test and the Begg test were used. However, funnel plots have come under criticism for gauging publication bias only [27] so other possible sources of asymmetry were considered. Finally a forest plot was drawn using 95% confidence limits under the random-effect model to summarise and conclude this study.

## Results

### Delimiting appropriate studies for meta-analysis

**Fig. 1:**
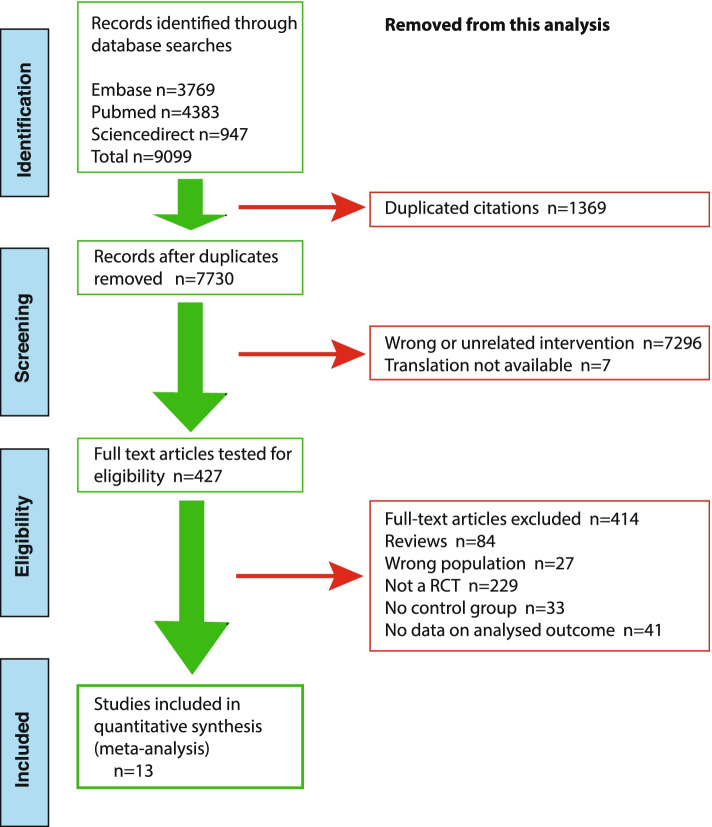
Overview of the hierarchical database search and subsequent screening procedure. Screening was performed to delimit studies for meta-analysis of peer-assisted learning in the medical curriculum using the PRISMA protocol; left-hand side of figure illustrating pipeline of articles selected and right-hand of figure the elimination of studies from an initial recovery of ~9100 articles through to the 13 studies selected for this study

This analysis is focused on investigating whether studies on peer-assisted learning show any consistent improvement in learning achievement of clinical skills and knowledge as measured by examination(s). Searching of the relevant databases, Pubmed, Embase and Science Direct, revealed numerous studies that have investigated the possible effects of peer-assisted learning. However, we wished to focus on those conducted in medical schools. Using a hierarchical search strategy, we identified peer-assisted learning studies using some form of randomized control trial. This initial screen yielded just over 9000 publications related to peer-assisted learning. Next, PRISMA protocols were used to further delimit those reports that adhered to a minimal set of reporting criteria to make them suitable for this meta-analysis and combined this with a risk of bias assessment as provided by Cochrane collaboration’s tool. An overview of the selection protocol to identify sufficiently robust, controlled and focused reports relevant to the aim of this study is provided in Fig. 1.

### Analysis of risks with selected studies

Thirteen studies were included for the meta-analysis [28–40] comprising a total sample size of* n *= 2,003 students. The nature and characteristics of the individual peer-assisted learning studies chosen is summarized in Table 1, and as demonstrated in this table, these originate from different areas of the clinical skills curriculum.

**Table 1:** Summary of characteristics of studies chosen for the clinical skills/knowledge meta-analysis

Lead author (Year)	Sample size (Intervention)	Sample size (Control)	Subject	Grade of student	Grade of peer tutor	Level of control teacher	Nature of intervention	Duration of intervention	Tutor training mentioned	Objective outcomes measured (examinations)	Location of study	Any statistical significance	Reference #
Büscher (2013)	63	60	Basic pediatric examination (newborns) and communication skills with parent	One year junior to tutors	Already completed semesters 4&5 (i.e. senior)	Senior lecturer	Student peer tutor teaching using checklist to groups of 2 students	14 days pediatrics class	2 weeks (80 hours)	OSCE (some videoed for expert validation)	Germany	No (*p* = 0.2)	[28]
Cremerius (2019)	36	33	Basic musculoskeletal ultrasound skills including shoulder	Elective from semester 4 onwards	Matched peer	Qualified doctor with several years ultrasound experience	Student peer tutor demonstrates patient positioning, device handling, 3 ultrasound views and explanations. Student practiced learnt skills with each other. Group size maximum 6.	one semester	2 weeks in advance	MCQ & OSCE	Germany	MCQ - no (*p *= 0.75); OSCE - no (*p *= 1)	[29]
Heckmann (2008)	66	56	Neurology (neurological examination and lumbar puncture simulation)	Year 5	Students who succesfully completed course one semester earlier (Year5/6)	Postgraduate tutors	Peer tutor instructs and demonstrates and subsequently oversees student practice. Groups 8-10.	one week	Briefed prior to course by neurologist (undefined further)	Written test & OSCE	Germany	Written test - no (*p* = 0.15); OSCE - no (*p* = 0.11)	[30]
Hudson (2008)	64	67	Basic clinical skills (medical history, patient examination and patient communication)	Year 2	Year 6	Paid doctors	Small-group structured tutorials for feedback on history taking and examination skills using simulated patient.	14 weeks including 14x 1 hour small group tutorials	Training workshop and continued educational support	OSCE	Australia	OSCE - no overall difference (only 1 of 6 stations had difference *p* < 0.05)	[31]
Kassab (2005)	44	47	Hematology unit (clinical knowledge) PBL Health problems	Year 3	Matched peer	Faculty tutor	2 tutorials/week. Group size 8-10 students.	5 weeks	Intensive one-day workshop and weekly tutor-briefing	End-unit exam: SAQ, MCQ & Observed Structural Practical Exam	Kingdom of Bahrain	SAQ and MCQ combined - no; OSPE - no; (*p*-values not given beyond >0.05)	[32]
Knobe (2010)	75	76	Musculoskeletal ultrasound of the shoulder (sports medicine)	Years 3 and 4	Matched peer	Experienced doctor >6 years	Two x 120 minute teaching sessions: theory (30 min) practice (90 min). Group size 6-14 students	Two weeks (2 hours/week)	30 minute training and 1 week self-teaching	MCQ & OCSE	Germany	MCQ - no (*p *= 0.64); OSCE - no (*p *= 0.13).	[33]
Kühl (2012)	15	15	Internal medicine (cardiology) - focused emergency echocardiography skills	Year 3-5	Year 3-6	Expert cardiographer	Three x 135 minute hands-on practical sessions. Student/instructor ratio 1:3.	Twelve hour course	5h seminar, 3 week practical, 3h expert meeting, 12h didactic seninar	OSCE	Germany	Yes – *p* = 0.03	[34]
Nomura (2017)	58	58	Communication skills - Medical interview training	Year 4	Year 5	Faculty physician	Small group role-play of a medical interview (3 hour session). Group size undefined	4 week course	One hour tutor training	OSCE	Japan	No (p value not given but did not exceed 95% CI non-inferiority magin)	[35]
Rogers (2000)	40	37	Computer-assisted learning of a surgical skill	Year 1	Matched peer	Not applicable (software)	Group size 8-10 where either 1 student:1 peer or individual student interacts with computer. Post session performance videotaped and validated by surgical faculty.	Undefined	Undefined (beyond computer assisted learning program for both)	Practical examination	USA	Yes but worse outcomes (*p* = 0.04)	[36]
Shah (2017)	60	60	Physical examination skills (surgery clerkship): examination of lump, limb, palpation and percussion of abdomen.	Year 5	Matched peer	Expert (no further definition)	One hour clinical skills teaching sessions. Group size 5.	4 weeks	Training given but extent not specified	OSCE	Pakistan	Yes – *p* = 0.00	[37]
Steele (2000)	64 (approx half of 127 cohort)	64 (approx half of 127 cohort)	PBL case studies (clinical knowledge)	Year 2	Matched peer	Faculty members	11 case studies: Each inviolves 3x 1-2h PBL sessions. Group size = 10.	11x 2 weeks	Pre-case orientation sessions	15 item exam following each case, MCQ and matching questions	USA	No (No *p*-value given)	[38]
Weyrich (2009)	31	28	Basic clinical skills - internal medicine technical skills	Year 3	Year 4 & 5	Consultants in internal medicine	Two training units (each 2x 3-hour training sessions delivered one week apart. Group size 5-8 with 2 tutors.	6 months	Two 3 hour skills training sessions & two 4-hour teaching sessions	OSCE	Germany	No (*p* = 0.11)	[39]
Widyahening (2019)	241	241	Clinical epidemiology and evidence-based medicine - critical appraisal skills	Year 4	Newly graduated doctors	Medical Staff	Four 2-hour tutored group discussions. Group size 10-11 students	4 weeks	3 day training of teachers	MCQ & Fresno test	Indonesia	No (*p* = 0.12)	[40]

**Fig. 2:**
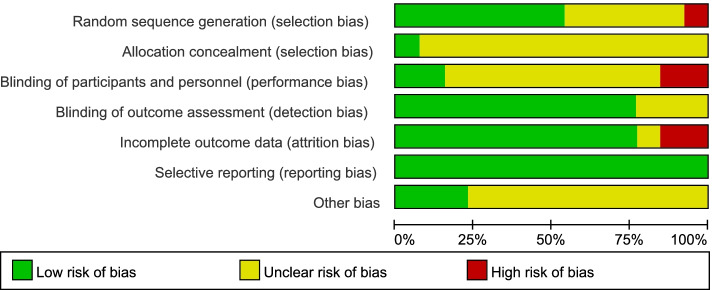
Risk assessment for bias for the overall group of studies selected. Risk was assessed according to Cochrane collaboration’s tool

**Fig. 3:**
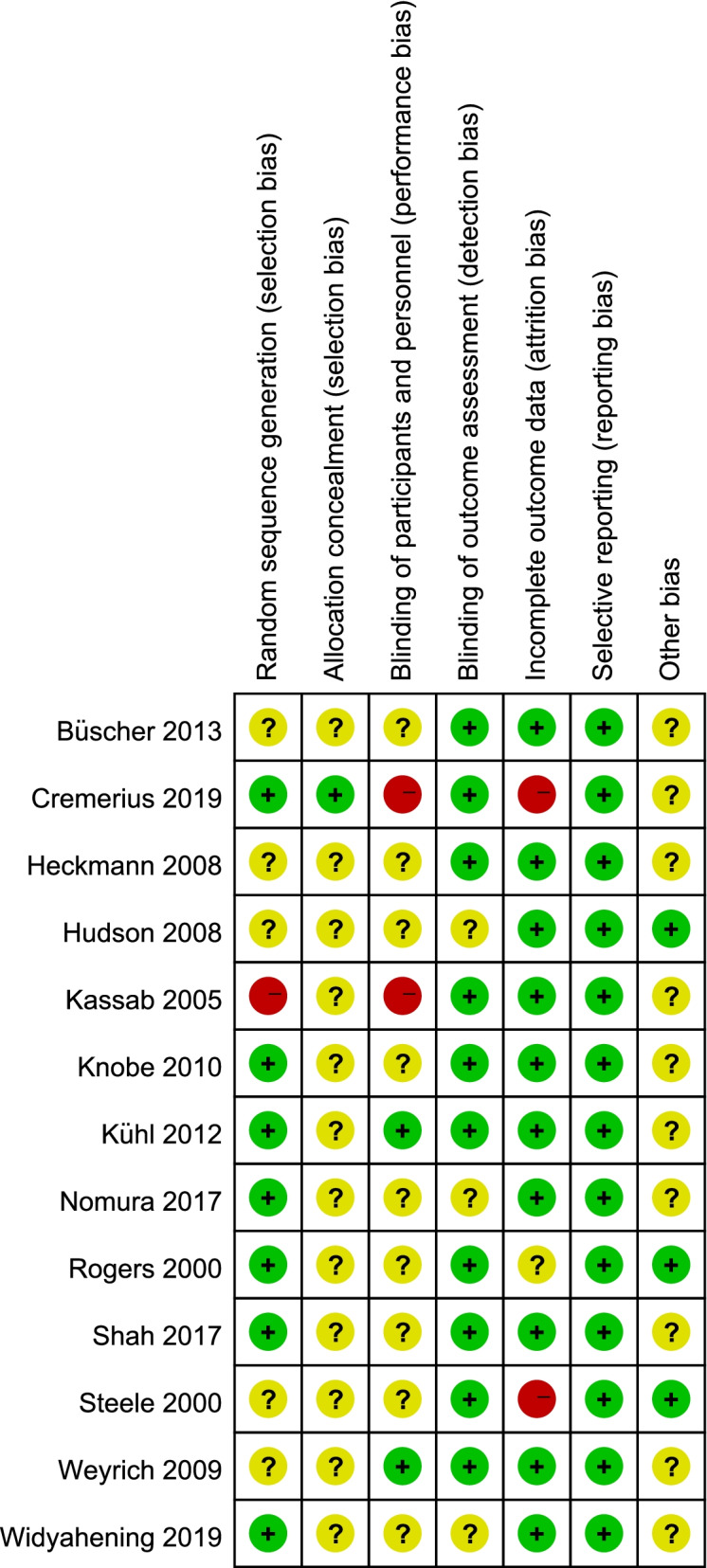
Risk of bias summary for the individual studies assessed. Risk was assessed according to Cochrane collaboration’s tool for assessing risk of bias; colours representing levels of bias are as indicated in Fig. 2

**Fig. 4:**
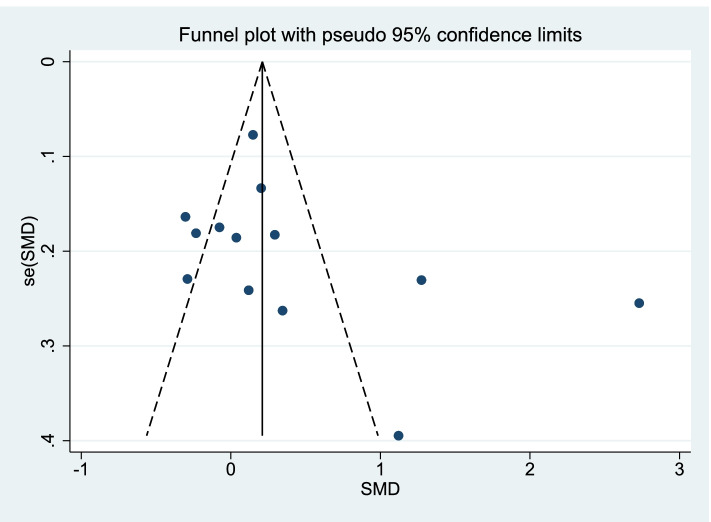
Funnel plot to investigate publication bias. Standard error was used as method to quantify study precision. Each dot represents a specific study; the y-axis represents study precision (SE-standard error) and the x-axis shows the study’s result (SMD- standardized mean difference)

**Fig. 5:**
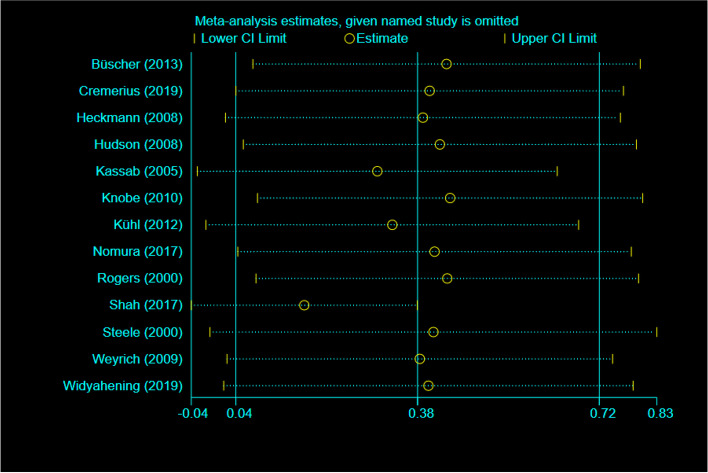
Between-study heterogeneity analysed by influence analysis. Influence analysis was carried out by using metaninf to investigate the influence of each individual study on the overall meta-analysis summary. The horizontal axis indicates the overall standard mean difference and the two vertical lines each end indicate 95% CL. Circles indicate the pooled effect calculation when the study as indicated on the left is omitted. This demonstrates the Shah (2017) study as an outlier

A risk analysis was subsequently carried out on the group of selected studies using Cochrane collaboration’s tool and is represented graphically in Fig. 2. This analysis indicated there was a low risk of reporting, detection and selection biases (Fig. 2). Only minimal levels (<20%) of high-risk bias was suggested from performance and attrition categories, although we note there were also varying levels of unclear risk of bias for all risk categories (Fig. 2). However, from the level of risk that was categorised using this tool, these studies used together appear to carry a relatively low risk of bias. Risk assessment of bias for the individual studies indicated only 3/13 studies showed a high level of risk bias in any category (Fig. 3), and the randomized controlled trials chosen were therefore judged to be of moderate to high quality. Whilst funnel plots have come under criticism for gauging publication bias only [27], visual inspection of the funnel plot comparing the standard error as a measure of study precision revealed asymmetry (Fig. 4). Tests for asymmetry in funnel plots are of low power, and since substantial heterogeneity was detected in this study, the number of studies required would need to be appreciably greater than the minimum recommended number of 10 studies [27] to be reliable. But although this meta-analysis only slightly exceeds this number, we performed two tests for publication bias. The Egger test was not significant for publication bias (*P* = 0.249), whereas the Begg test was (*P *= 0.033). However, when the number of studies is low, the efficiency and reliability of the Egger test using linear regression is believed to be more powerful than the Begg method, that uses rank correlation, and has very low power to detect biases for small sample sizes [41]. The asymmetry in the funnel plot could therefore suggest some reporting bias, which may be explained by studies that show less favourable effects not being published, compared to studies that do show favourable effects being more likely to be both written up and accepted for publication. There are several other possible explanations in addition to reporting bias that can lead to funnel plot asymmetry, such as heterogeneity, methodological quality and chance. Using the random effect model, if heterogeneity was large the plot would appear cylindrical [27] which it does not (Fig. 4). To analyse heterogeneity further an influence analysis was carried out. Between-study heterogeneity was examined where pooled estimates were calculated repeatedly, but omitting one study in each successive calculation (Fig. 5). This revealed that one study (Shah, 2017, [37]) had a very high effect size and was a clear outlier, and the overall effect size of pooled studies is smallest when this study is removed. Thus this study may be the main source of heterogeneity and distort the effect size estimate. The methodology used in studies could also lead to asymmetry, but in the selected studies, all appeared well set up and statistically analysed. Finally, the role of chance in leading to the observed plot asymmetry cannot be ruled out given the relatively small number of studies and the heterogeneity detected. However, taking into account the above caveats, this asymmetry could also indicate that the intervention of peer-assisted learning is indeed having a positive effect on objectively assessed learning outcomes.

### Meta-analysis of the selected studies

**Fig. 6:**
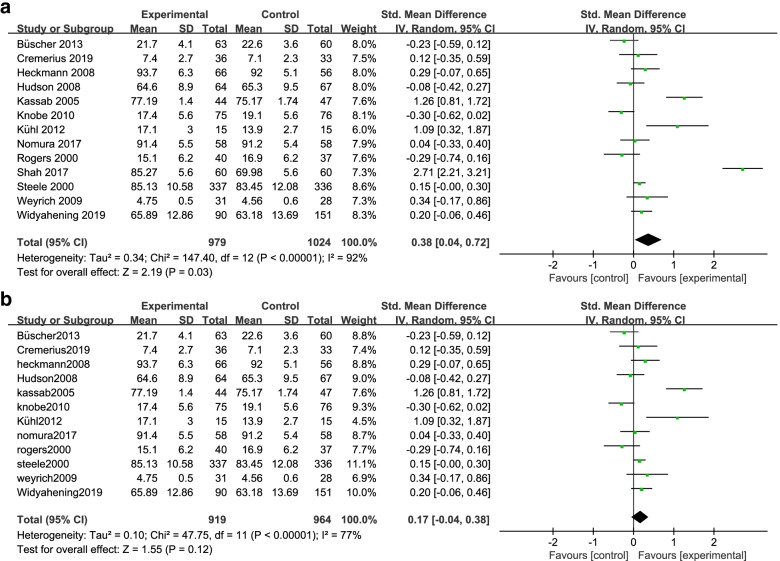
Statistical meta-analysis and forest plot illustrating the effect of peer-assisted learning. In the forest plot, the pooled effect (solid diamond) summarizes the meta-analysis revealing a marginal but overall improvement in learning (Favours – experimental) using peer-assisted learning considering all studies together. Individual studies vary in their outcomes, but the majority either exhibit improvement, or little or no detriment to medical student learning. **A** Analysis of all 13 selected studies. **B** Analysis of studies omitting the study of Shah (2017)

The meta-analysis investigating whether treatment (peer-assisted learning) leads to any difference compared to controls is graphically presented as the forest plot in Fig. 6, either including (Fig. 6A) or excluding (Fig. 6B) the Shah (2017) study. Considering all 13 studies together (Fig. 6A), the meta-analysis indicated that medical students using peer-assisted learning showed an enhanced examination performance compared with traditional teacher-led learning overall. This result is statistically significant (*P *= 0.03) with significantly high heterogeneity (*I²*=92%). The Shah study was shown to be a significant outlier above, therefore the forest plot was recalculated omitting this study since it is particularly influential as one of only three individual studies showing statistically significant improvement in assessed outcomes of peer-assisted learning (Fig. 6A). Removing this study from the meta-analysis reduces heterogeneity of the analysis (*I²*=77%), but the overall effect of the interventions no longer remains statistically significant (*P *= 0.12) (Fig. 6B). The forest plot illustrates that analysis of the 12 pooled studies showed there is no detriment to assessed outcomes from peer-assessed learning (Fig. 6B). Thus peer-assisted learning is not inferior to teacher-led learning. However, there is no longer a statistically significant improvement in the assessed outcomes considering the pooled studies. Further, individually, only two of the 12 studies show improvement of assessed outcomes at a statistically significant level (Fig. 6B).

## Discussion

### Meta-analysis of peer-assisted learning in clinical skills and knowledge

This analysis of the pooled effect of peer-assisted learning compared to teacher-led learning in a set of clinical skills and knowledge subjects revealed there was no detriment to examination performance as an objective measure of learning outcome. For two of the clinical subjects, the haematology unit (clinical knowledge) and emergency echocardiography (skills), the improvement of peer-assisted learning over teacher-led learning was statistically significant (95%CL). Considering the other individual studies however, there was no statistically significant enhancement of assessed learning outcome, although as for the pooled effects, peer-assisted learning was not detrimental, an important finding as class sizes become larger as the demand for more trained doctors increases. This analysis therefore supports the notion that peer-assisted learning in clinical skills is a useful form of active experiential learning, and furthermore, can enrich the student experience by providing additional variety in teaching and learning modes to accommodate the different learning preferences that students bring.

Are there any commonalities between the two studies [32, 34] that show statistically significant improvement that may explain why these studies appear to offer enhanced learning outcomes? The specific subject matter does not appear critical as they come from different areas of clinical skills and knowledge training. Also, the academic level of the peer-tutor varies involving either horizontal learning, learning from fellow student peers at the same educational level, or vertical learning involving student peer tutors at nearby but more advanced academic levels. In both studies peer-tutor training was provided, albeit to differing extents, but this is also true of studies that were found not to exhibit statistically significant improvement in assessed learning outcome. The parameter that may influence the success of peer-assisted learning is the academic level of student learners from at least year 3. By year 3, students should have developed better educational maturity in a higher education setting that involves less didactic teaching and have become more receptive to learn from their peers and near-peers. However, better educational maturity is likely to be only one contributory factor in addition to student and student-tutor motivation, complexity of subject matter, effectiveness of tutor-training and factors that are discussed below. It is worth bearing in mind that one study (Shah et al.) was identified as a possible cause of heterogeneity. When eliminated from the final meta-analysis, the overall meta-analysis no longer showed a statistically significant improvement in assessed outcome but did indicate no detriment to learning. This warns against over-interpretation of individual studies. Nevertheless, the statistical exclusion of just one from 13 studies indicates there is merit in considering the mechanisms involved, advantages and disadvantages of incorportating peer-assisted learning in the medical curriculum.

### How peer-assisted learning may contribute to learning

Ten Cate and Durning [42] put forward the cognitive congruence hypothesis to describe how peer-assisted learning can have a positive impact when studying with peers of a similar educational level. Knowledge gaps amongst peers may be better understood than in teacher-directed learning as there is a similar level of baseline knowledge. This is in contrast with teacher-led learning, where the teacher may make incorrect assumptions concerning existing knowledge of fundamental concepts, particularly when teaching elements at a higher educational level where these concepts are critical for understanding. This knowledge gap could influence the motivation of students to learn.

The cognitive congruence hypothesis suggests that in a peer-peer learning community, students will be more open and less guarded when they get along with their peers. However, this indicates that the relationship between students also affects the group learning state. This could create inequalities in the effectiveness of peer-assisted learning. Differences in individual students’ study habits and their social competence can also impact on them being receptive and open to learning from peers, and gender biases may also influence peer-assisted learning [43].

### Student concerns with peer-assisted learning

Students involved in peer-assisted learning retain concerns about the depth of knowledge and clinical experience of the peer-tutor to answer technically complex questions [11], and that peer tutors cannot compete with professional teachers with better pedagogical skills. Further, student ideology may be influenced by the reality of paying for a “traditional” medical education, and the negative perception engendered by receiving that education from fellow students. However, learning achievements with peer-tutors and professional teachers appear comparable when learning straightforward elements of knowledge, but the value of teacher-led learning appears to increase alongside the difficulty of the subject [38]. Since students worry about making mistakes in their learning that are not identified by their peers, it was suggested that esoteric and complex content is best taught by professional teachers and experts [38]. This meta-analysis and others across higher education provide evidence of the gains in student learning and should help counteract the variety of student concerns.

### Benefits of peer-assisted learning

Peer-assisted learning serves as an important form of active learning that can improve knowledge retention and metacognitive awareness. During peer-assisted learning, students are less fearful of making errors in front of their peers rather than faculty, and therefore will have more opportunities to identify their defects and correct them before examinations and qualification. This promotes the development of clinical skills, where continued practice is effective in building and honing basic skills.

Students also have different learning preferences that relate to their individual strengths and weaknesses within different learning styles [44], and although learning styles and their use in educational strategies has attracted some criticism (reviewed in [45]), it is undoubtedly the case that students’ learning abilities and strategies are not homogenous. We would argue that any educational strategy that adopts a variety of teaching methods is likely to benefit and be more inclusive to a wider proportion of the student cohort. It is not envisaged that peer-assisted learning would replace teacher-led learning, but rather that when used as a supplementary tool in the appropriate context, it has excellent potential to complement traditional teacher-led activities in the curriculum [10].

There are additional benefits of peer-assisted learning for the peer tutor. Peer-assisted learning is a bidirectional, reciprocal process, where mutual benefit is at the core [9, 46, 47], and as any educator discovers when they first begin teaching, it is only when you attempt to explain to others that you fundamentally appreciate the level of your own understanding. This interplay between students as peer-learner and peer-tutor creates a more comfortable and less hierarchical teaching environment, making learning easier and more readily accessible [11, 30, 31, 35, 47] and helps towards building students’ confidence [11, 14, 16, 31, 32, 35, 40, 48]. Since academic goals are shared in this collaborative learning effort, this promotes deeper learning and reduces stress during the learning process [30, 38], where students are more engaged and can have greater ownership over their knowledge acquisition.

### The case for peer-assisted learning in the medical curriculum

Medical students are a unique group within higher education as early in the medical curriculum, students are required to acquire a vast volume of fundamental theoretical knowledge that later aligns to varying degrees with its application to clinical practice. This overwhelming amount of knowledge unfortunately encourages memorization as a learning strategy for many students, leaving little room for deeper understanding and higher order thinking, ultimately leading to superficial learning. However, as the medical curriculum progresses, a stronger link of theoretical knowledge with application emerges, as students are exposed to clinical practice including hospital rotations and primary care. Strong “hands-on” skills are developed as well as the translation of theory into practice, and at this later stage, rote learning following didactic teaching approaches is insufficient to provide the knowledge required.

In medical school curricula in many countries, an ability to effectively communicate with others, from patients and their families through to other health care professionals is seen as an important competency [40]. Peer-teaching allows students to develop their conversational skills in a safe environment that will improve their communication competency [40].

Thus compared with traditional lectures, peer-assisted learning would seem to be much more aligned with medical students’ needs of applying theory and practice through active learning, “doing” rather than memorizing. Beyond the obvious benefits of learning per se, peer-assisted learning builds up self-confidence and collaborative skills for learning, and the ability to teach, lead and listen within a team. Together these skills will become important attributes for the student in future clinical practice.

### Strengths and limitations

This study used meta-analysis protocols to generate a more accurate estimate of the effect of peer-assisted learning by analysing pooled studies rather than any one individual study alone [49]. Literature included in this study were all randomized controlled trials and variants of these such as randomized crossover studies, providing individual studies of high-quality, and their use of blinding (Figs. 2 and 3) helps reduce bias in the overall result.

This meta-analysis was limited to English-language publications, and qualitative research and grey literature were excluded. Furthermore, some heterogeneity was demonstrated in this meta-analysis and discussed above. It is not possible to conclude that peer-assisted learning is always “better” or more “successful” than traditional teacher-led activities. Instead, the meta-analysis is sufficiently robust to suggest that peer-assisted learning is an effective tool for targeted use in the medical curriculum in clinical skills learning with an effect that can be at least comparable, and sometimes an improvement, to traditional teacher-led learning.

An uncontrolled factor in studies of peer-assisted learning is likely to be the quality, training and motivation of peer tutors, factors that are likely to vary from tutor to tutor and institution to institution. Whilst difficult to control, addressing inequality in individual peer-tutor ability and motivation is likely to be key to the successful use of peer-assisted learning. In addition, students need to “buy in” to peer-assisted learning for this approach to be successful.

## Conclusions

This study demonstrated that peer-assisted learning is not detrimental to student learning as assessed by examination performance compared with teacher-led learning. Thus peer-assisted learning can aid in the development of a useful community of learning in clinical skills and clinical knowledge, with the caveats of ensuring the appropriate level of complexity in the learning task, and the appropriate training of peer tutors. However further studies into peer-assisted learning are required. For example, since peer-assisted learning can improve assessment performance in medical students’ clinical skills and knowledge education in some cases, the parameters that lead to this remain unresolved but could be addressed through more high-quality and focused randomized control trials.

## Data Availability

The individual datasets were taken from the publications cited in Table 1, and the data generated and analysed included in this article.
